# Prognostic implications of venous invasion in pancreatic cancer following radical surgical resection: a pathological perspective

**DOI:** 10.3389/fmed.2025.1613153

**Published:** 2025-07-23

**Authors:** Jin-Can Huang, Cheng-Run Zhang, Han-Xuan Wang, Shao-Cheng Lyu, Ren Lang, Tao Jiang

**Affiliations:** Department of Hepatobiliary, Pancreas & Spleen Surgery, Beijing Chao-Yang Hospital, Capital Medical University, Beijing, China

**Keywords:** pancreatic cancer, venous invasion, pathology, prognosis, pancreaticoduodenectomy

## Abstract

**Background:**

The assessment of resectability in borderline resectable pancreatic cancer (BRPC) primarily relies on preoperative imaging, which examines the spatial association between the tumor and adjacent vital veins, particularly the portal venous system. However, venous invasion detected via imaging is rarely confirmed by postoperative pathology. This research seeks to assess the long-term results in patients with pathologically verified venous invasion and to explore the related risk factors.

**Methods:**

This study involved 248 individuals who underwent radical pancreaticoduodenectomy (PD) from January 2011 to July 2023. Patients were classified into three categories based on preoperative imaging and postoperative pathology: no venous invasion (*n* = 99), imaging-suspected (*n* = 30), and pathology-confirmed (*n* = 119). Clinical features and prognoses were compared among groups. In the cohort with pathology-confirmed venous invasion, patients were further classified by invasion depth into intimal (*n* = 51) and non-intimal (*n* = 68) subgroups for risk factor analysis.

**Results:**

Patients with pathologically confirmed venous invasion exhibited significantly reduced disease-free survival (DFS) and overall survival (OS) when compared to those with either imaging-suspected or absent venous invasion (*p* < 0.001). Further analysis of pathology-confirmed venous invasion patients showed significantly lower DFS (*p* = 0.008) and OS (*p* < 0.001) compared to non-intimal invasion patients. Independent risk variables for poor DFS included age ≥60 years, tumor diameter >3 cm, and intimal invasion. Independent risk factors for poor OS in patients with venous invasion included age ≥60 years, poorly differentiated tumors, intimal invasion, and TNM stage III.

**Conclusion:**

Intimal venous invasion emerged as a distinct risk factor influencing postoperative survival. Radical surgical resection remains essential for attaining favorable long-term outcomes in pancreatic cancers.

## Introduction

Pancreatic carcinoma, being a malignancy of high aggressiveness, has seen an increasing incidence and mortality rate globally and has a mere 8% five-year survival rate (1, 2). Due to its unique anatomic location, pancreatic cancer easily invades the adjacent portal venous system. Additionally, patients often exhibit subtle symptoms, and 80% are already in the locally advanced stage at the time of diagnosis, further increasing the difficulty of surgical resection (3). Although chemotherapy and immunotherapy have rapidly advanced in cancer treatment over the past decades, their efficacy in pancreatic cancer remains limited (4–6). Radical surgical resection remains essential for attaining favorable long-term outcomes in pancreatic cancers.

Pancreatic neoplasms are stratified into three distinct surgical feasibility categories—“Resectable,” “Borderline Resectable,” and “Locally Advanced”—through systematic evaluation of anatomical interactions between malignant masses and critical vascular structures in the abdominal region (7). Extensive evidence has demonstrated the safety and effectiveness of radical PD combined with venous resection and reconstruction (8, 9). While neoadjuvant therapy can facilitate downstaging in certain BRPC patients, the conversion rate for neoadjuvant sequential therapy ranges from 12 to 27% (10, 11). Certain patients with BRPC may still achieve a surgical cure at specialized pancreatic centers by upfront surgery accompanied by venous resection and reconstruction.

Currently, the definition of BRPC is predominantly reliant on preoperative imaging to assess the interaction between the pancreatic tumor and surrounding venous structures, hence defining the degree of venous invasion to establish the cancer’s resectability. Nonetheless, a certain level of false positives persists in the diagnosis of BRPC, even among proficient radiologists or pancreatic surgeons. Pathology, regarded as the gold standard, is hardly utilized in assessing the degree of vascular invasion in pancreatic ductal adenocarcinoma (PDAC) patients. The present research conducted a retrospective analysis of the clinical data from individuals diagnosed with pancreatic carcinoma, integrating preoperative imaging assessments and postoperative pathology findings, to further explore the predictive outcomes of individuals receiving radical resection.

## Materials and methods

### Patient selection

**Figure 1:**
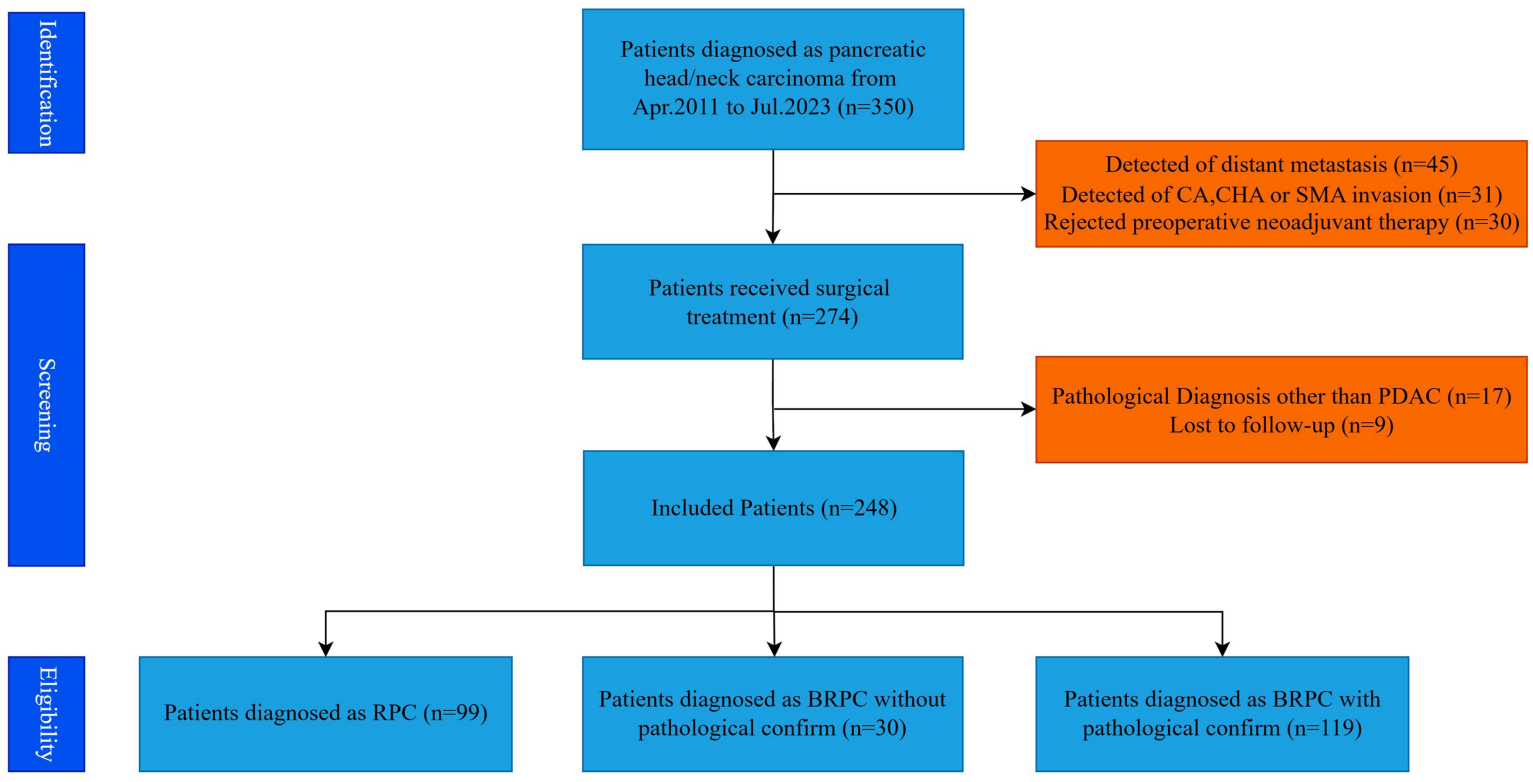
Criteria for participant inclusion and exclusion in the study.

A retrospective analysis was performed on the clinical data of 248 pancreatic cancer patients who underwent radical PD at the Department of Hepatobiliary Surgery, Beijing Chaoyang Hospital, Capital Medical University, from Jan 2011 to July 2023. The standards for patient inclusion and exclusion in this particular study are depicted in Figure 1. Criteria for inclusion: (1) Preoperative imaging identifies resectable or borderline resectable pancreatic cancer, as defined by the 2014 National Comprehensive Cancer Network (NCCN) guidelines (7); (2) Tumor situated in the pancreatic head or neck; (3) Postoperative pathology confirms pancreatic adenocarcinoma; (4) Informed consent for the surgical procedure was secured from the patient and their family. Exclusion criteria: (1) Administration of neoadjuvant therapy before surgery; (2) Intraoperative identification of vascular invasion; (3) Intraoperative identification of distant metastases; (4) Loss to follow-up. The research adhered to the Declaration of Helsinki (2013 revision) and received approval from the Ethics Committee at Beijing Chao-Yang Hospital (No. 2021-D-16) for the use of allogeneic venous vascular replacement procedures; as a retrospective study, it was exempt from informed consent requirements.

### Patient grouping and definition

Patients were categorized according to preoperative imaging and postoperative pathology findings. Ninety-nine patients had radical PD, with preoperative imaging and postoperative pathology indicating the absence of venous invasion, and were categorized into the no venous invasion group (Group 1, *n* = 99). Thirty patients had radical PD together with resection and repair of the involved vein. Preoperative imaging indicated potential invasion of the portal venous system; however, postoperative pathology did not substantiate venous invasion, resulting in classification into the imaging-suspected venous invasion group (Group 2, *n* = 30). One hundred and nineteen patients had radical PD together with resection and repair of the involved vein. Preoperative imaging revealed invasion of the portal venous system, and postoperative pathology corroborated venous invasion, so categorizing it into the pathology-confirmed venous invasion group (Group 3, *n* = 119). Patients with pathology-confirmed venous invasion were classified into two subgroups depending on the level of invasion: the intimal invasion group (*n* = 51) and the non-intimal invasion group (*n* = 68).

### Surgical strategy

**Figure 2:**
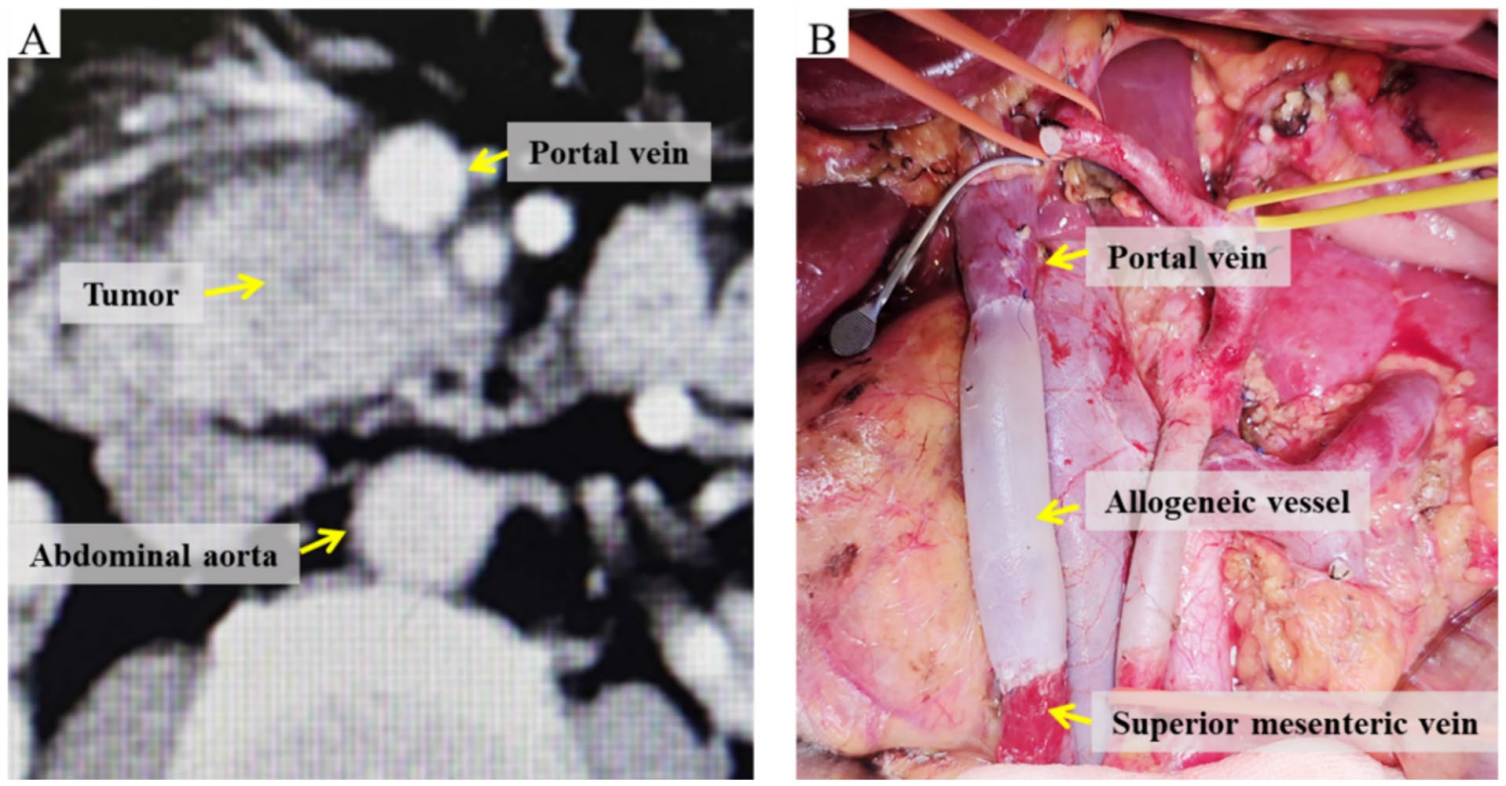
Images of the involved vein before and after radical PD with venous resection and reconstruction. **(A)** Preoperative CT demonstrating tumor invasion of the portal vein. **(B)** Intraoperative view of allogeneic vein graft reconstruction.

All patients underwent complete tumor excision during surgery, succeeded by radical PD with lymphadenectomy. In individuals suspected of having venous invasion associated with pancreatic cancer (Figure 2A), following the resection of the invaded vein. Vascular reconstruction may be executed by directly suturing the vein with 6-0 or 7-0 Prolene vascular sutures, therefore maintaining vascular tension and hemodynamic stability. Alternatively, preserved allogenic vessels may be employed, adjusted to the requisite length and configuration, and sutured at both extremities using 6-0 or 7-0 Prolene vascular sutures. The posterior and anterior walls are constantly sutured to finalize the anastomosis between the recipient and allogenic vessels (Figure 2B). Postoperatively, low-molecular-weight heparin is supplied to avert venous thrombosis in the repaired vein, contingent upon the exclusion of postoperative bleeding risk.

### Follow-up strategy

Patients underwent re-evaluation at 1 month and 3 months post-surgery, followed by assessments every 3 months during the initial 2 years after the operation. If the results remained stable, the follow-up frequency could be adjusted to once a year. Follow-up evaluations comprised blood tests and imaging procedures, incorporating contrast-enhanced computed tomography (CT) imaging of the abdomen and thorax. OS was defined as the duration from the date of surgery to either the date of death or the last follow-up, while DFS was defined as the time from the surgery date to the identification of local recurrence, metastasis, or the last follow-up assessment. The follow-up period ended in July 2024, with the primary endpoint being patient death.

### Statistical analysis

Continuous data were expressed as either the mean ± standard deviation (SD) or the median with interquartile range (IQR). For categorical variables, chi-square or Fisher’s exact tests were employed. Survival outcomes were evaluated using Kaplan–Meier curves, supplemented by log-rank tests. Univariate and multivariate analyses were conducted using the Cox proportional hazards model to determine independent prognostic indicators. A *p*-value of less than 0.05 was considered statistically significant.

## Results

### Baseline and clinical features of patients

This study included a cohort of 248 consecutive patients, comprising 147 males and 101 females, with a mean age of 62.9 ± 10.0 years. The primary symptoms at the onset for these patients were as follows: jaundice was reported in 119 cases, abdominal pain in 87 cases, and non-typical gastrointestinal symptoms in 12 cases. Additionally, a diabetic history was identified in 82 participants (33.1%). Among those presenting with jaundice, 67 received preoperative treatments aimed at reducing jaundice, which included endoscopic retrograde cholangiopancreatography (*n* = 10) and percutaneous transhepatic biliary drainage (*n* = 57).

As is shown in Tables 1, 2, compared to patients in Group 1, a higher proportion of patients in Groups 2 and 3 had a history of smoking, with *p*-values of 0.01 and 0.023, respectively. Concurrently, as observed in Table 2, due to the combined resection and reconstruction of involved veins, patients in Groups 2 and 3 required longer operative times and experienced greater intraoperative blood loss compared to Group 1, with *p*-values less than 0.05. Although the overall comparison in Table 1 revealed significant differences in CA19-9 levels and tumor diameter among the three groups, pairwise comparisons indicated that preoperative CA19-9 levels showed notable variations between individuals in Groups 2 and 3, and significant differences in tumor diameter between patients in Groups 1 and 3, as well as between Groups 2 and 3.

**Table 1:** Comparison of general information among three groups.

Variables	Total (*n* = 248)	Group 1 (*n* = 99)	Group 2 (*n* = 30)	Group 3 (*n* = 119)	*p*-value
Gender (male/female)	147/101	65/34	13/17	69/50	0.086
Age (year)	62.9 ± 10.0	64.4 ± 10.1	61.2 ± 9.4	6.0 ± 9.9	0.811
History of diabetes (yes/no)	82/166	32/67	8/22	42/77	0.655
History of smoking (yes/no)	80/168	42/57	5/25	33/86	0.010
Preoperative albumin (g/L)	37.3 ± 5.0	35.7 ± 4.9	38.3 ± 4.5	38.3 ± 4.8	0.679
Preoperative total bilirubin (μmol/L)	46.6 (12.7, 142.8)	85.7 (20.1, 178.9)	14 (8.1, 125.8)	25.7 (11.2, 130)	0.278
Preoperative CA19-9 (U/mL)	155.5 (39.4, 556.4)	145.4 (45.2, 481.5)	73.5 (21.3, 287.7)	208.9 (41, 774.1)	0.010
Preoperative drainage for jaundice (yes/no)	67/181	35/64	6/24	26/93	0.054
Intraoperative blood loss (mL)	500 (400, 800)	500 (400, 600)	600 (400, 800)	500 (400, 1,000)	0.000
Operation time (h)	11 (9, 11)	9 (8, 11)	12 (11, 13.2)	12 (11, 15)	0.001
Tumor size (cm)	3.2 (2.5, 4)	3 (2, 4)	3 (2.5, 3.9)	3.5 (3, 4.5)	0.022
Tumor differentiation (low/medium-high)	90/158	31/68	10/20	49/70	0.301
Pancreatic resection margin (R0/R1)	231/17	93/6	29/1	109/10	0.569
Lymph node invasion (yes/no)	168/80	62/37	18/12	88/31	0.128
Nerve invasion (yes/no)	243/5	96/3	30/0	117/2	0.549
TNM stage (I–II/III)	189/59	78/21	23/7	88/31	0.704
Postoperative complications (yes/no)	79/169	28/71	9/21	42/77	0.528
Postoperative chemotherapy (yes/no)	142/106	61/38	21/9	60/59	0.081

**Table 2:** Further comparison of general information between different groups.

Variables	Group 1 (*n* = 99)	Group 2 (*n* = 30)	Group 3 (*n* = 119)	*p*_12_-value	*p*_13_-value	*p*_23_-value
History of smoking (yes/no)	42/57	5/25	33/86	0.010	0.023	0.214
Preoperative CA19-9 (U/mL)	145.4 (45.2, 481.5)	73.5 (21.3, 287.7)	208.9 (41, 774.1)	0.065	0.297	0.017
Intraoperative blood loss (mL)	500 (400, 600)	600 (400, 800)	500 (400, 1,000)	0.045	0.000	0.386
Operation time (h)	9 (8, 11)	12 (11, 13.2)	12 (11, 15)	0.000	0.000	0.326
Tumor size (cm)	3 (2, 4)	3 (2.5, 3.9)	3.5 (3, 4.5)	0.377	0.000	0.047

### Survival outcomes of patients in three groups

In this study, the median duration of DFS was 15 months, with DFS rates of 55.4, 34.4, and 22.4% at 1, 2, and 3 years, respectively. The median OS duration was 18 months, with OS rates following radical surgery of 67.4, 37.6, and 23.4% at 1, 2, and 3 years, respectively.

**Figure 3:**
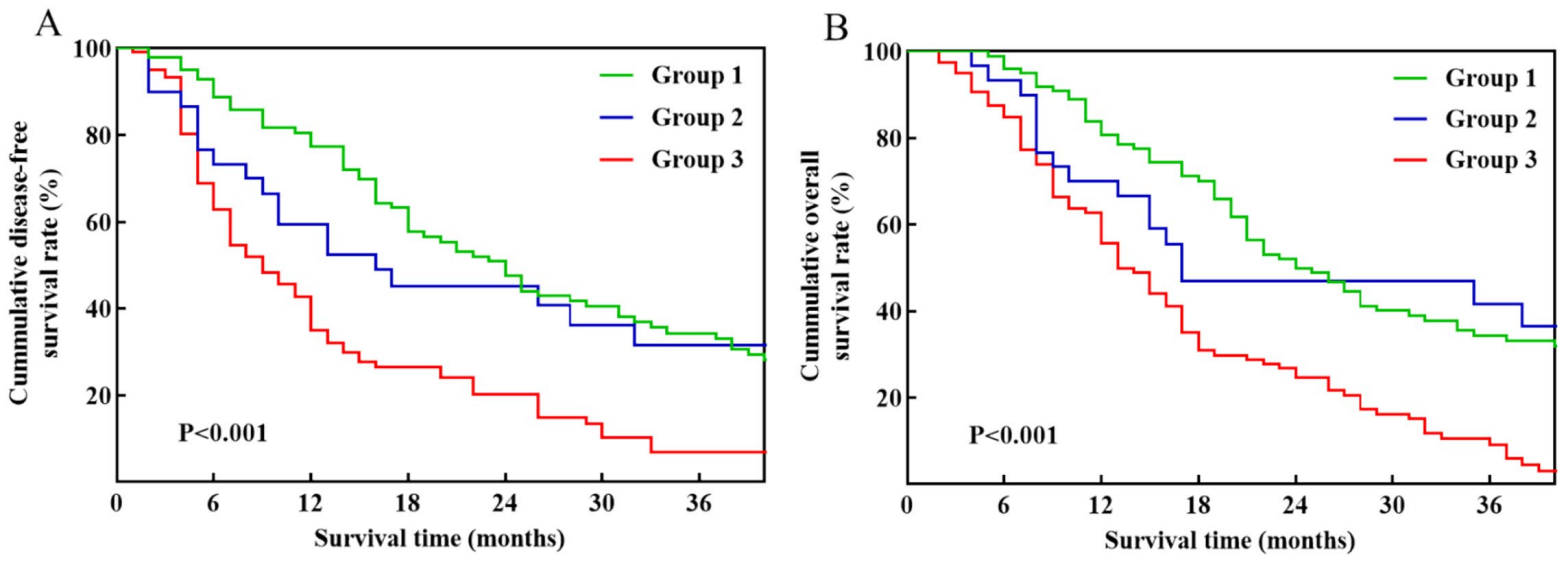
Kaplan–Meier survival curves among pancreatic cancer patients stratified by venous invasion status: Group 1 (no venous invasion), Group 2 (imaging-suspected venous invasion), and Group 3 (pathology-confirmed venous invasion). **(A)** DFS. **(B)** OS.

In the Kaplan–Meier analysis of DFS and OS based on the three groups, patients in Group 3 displayed significantly worse DFS (Figure 3A) and OS (Figure 3B) compared to those in Group 1 and Group 2 (*p* < 0.001). The median DFS for patients in Group 1 and Group 2 was 21 and 16 months, with corresponding 1-, 2-, and 3-year DFS rates of 77.4, 47.5, 34.4, and 59.5%, 45.2, 31.7%, respectively. The median OS for the two groups was 24 months and 17 months, respectively. The OS rates at 1, 2, and 3 years were 80.7, 50.0, and 34.4% for the Group 1, and 70.0, 46.9, and 41.7% for the Group 2.

**Figure 4:**
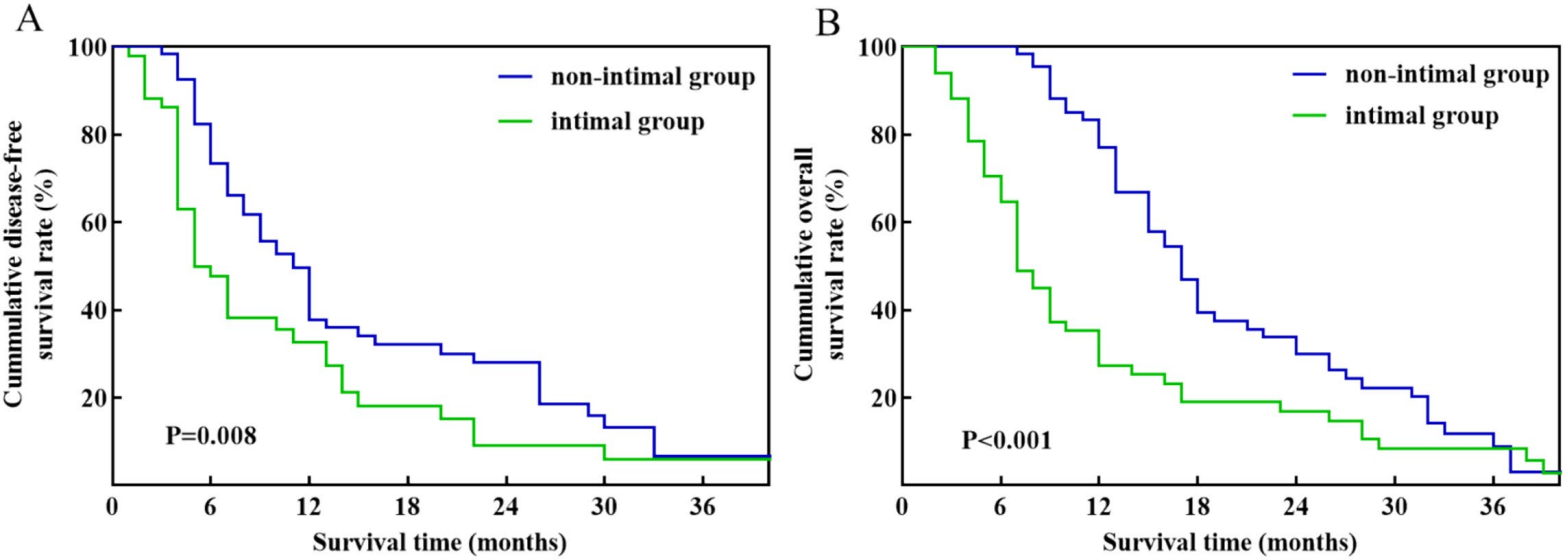
Kaplan–Meier survival curves between the intimal group and non-intimal group patients with pathology-confirmed venous invasion. **(A)** DFS. **(B)** OS.

Table 3 illustrates that there are no notable variations were observed in clinical and pathological characteristics between the intimal and non-intimal groups. Among patients with pathology-confirmed venous invasion, those in the non-intimal group demonstrated improved DFS (*p* = 0.008) and OS (*p* < 0.001) compared to their counterparts in the intimal group (Figure 4). The median DFS and OS for the intimal group were 9 months and 13 months, respectively.

**Table 3:** Demographic and pathologic findings in patients with pathology-confirmed venous invasion.

Variables	Non-intimal group (*n* = 68)	Intimal group (*n* = 51)	*p*-value
Gender (male/female)	39/29	30/21	0.872
Age (year)	63.3 ± 9.4	60.3 ± 10.5	0.145
History of diabetes (yes/no)	29/39	12/38	0.053
History of smoking (yes/no)	15/53	18/33	0.110
Preoperative albumin (g/L)	37.7 ± 4.6	39.2 ± 5.0	0.209
Preoperative total bilirubin (μmol/L)	24.7 (11.1, 128.7)	45.7 (11.2, 137.7)	0.749
Preoperative CA19-9 (U/mL)	179.8 (41.5, 542.1)	451.5 (37.7, 2004.7)	0.094
Preoperative drainage for jaundice (yes/no)	16/52	10/41	0.608
Intraoperative blood loss (mL)	500 (400, 1,000)	800 (500, 1,000)	0.127
Operation time (h)	11 (10, 14)	13 (11, 15)	0.124
Tumor size (cm)	3.5 (2.6, 4.5)	3.5 (3, 5)	0.120
Vein resection (end to end anastomosis/allogeneic graft)	25/43	10/41	0.042
Tumor differentiation (low/medium-high)	24/44	25/26	0.132
Pancreatic resection margin (R0/R1)	61/7	48/3	0.391
Lymph node invasion (Yes/No)	48/20	40/11	0.335
Nerve invasion (yes/no)	66/2	51/0	0.217
TNM stage (I–II/III)	53/15	35/16	0.252
Postoperative complications (yes/no)	22/46	20/31	0.438
Postoperative chemotherapy (Yes/No)	41/27	19/32	0.013

### Risk factors associated with prognosis in patients with pathology-confirmed venous invasion

In univariate Cox regression, intimal invasion, age, tumor differentiation, and TNM stage were significant for DFS (*p* < 0.05). In the multivariate Cox regression of DFS, age (HR: 2.145; 95% CI: 1.374–3.350; *p* = 0.001), degree of differentiation (HR: 1.607; 95% CI: 1.064–2.428; *p* = 0.024), intimal invasion (HR: 1.734; 95% CI: 1.145–2.652; *p* = 0.010), and TNM stage (HR: 1.670; 95% CI: 1.059–2.634; *p* = 0.027) were independent risk factors (Table 4).

**Table 4:** Univariable and multivariable analysis of the risk factors for DFS.

Variables	Univariate analysis		Multivariate analysis	
	HR (95% CI)	*p*-value	HR (95% CI)	*p*-value
Gender (male vs. female)	1.065 (0.708–1.603)	0.761		
Age (≥60 vs. <60 years)	1.872 (1.212–2.891)	0.005	2.145 (1.374–3.350)	0.001
History of smoking (yes vs. no)	1.017 (0.638–1.619)	0.944		
History of diabetes (yes vs. no)	0.847 (0.554–1.294)	0.442		
Albumin (<40 vs. ≥40 g/L)	0.733 (0.478–1.122)	0.152		
Total bilirubin (>26 vs. ≤26 μmol/L)	0.951 (0.632–1.429)	0.807		
CA19-9 (≥37 vs. <37 U/mL)	1.190 (0.718–1.971)	0.500		
Preoperative drainage for jaundice (yes vs. no)	1.088 (0.674–1.756)	0.730		
Blood loss (≥400 vs. <400 mL)	1.549 (0.986–2.432)	0.057		
Operation time (≥10 vs. <10 h)	1.196 (0.722–1.981)	0.487		
Degree of differentiation (poor vs. moderate-well)	1.664 (1.105–2.504)	0.015	1.607 (1.064–2.428)	0.024
Tumor size (>3 vs. ≤3 cm)	1.235 (0.820–1.859)	0.312		
Resection margin (R1 vs. R0)	1.135 (0.549–2.345)	0.733		
Vein resection (end to end anastomosis vs. allogeneic vein)	1.071 (0.683–1.679)	0.766		
Lymph node invasion (yes vs. no)	1.267 (0.800–2.008)	0.313		
Intimal invasion (yes vs. no)	1.681 (1.116–2.532)	0.013	1.743 (1.145–2.652)	0.010
TNM stage (III vs. I–II)	1.579 (1.010–2.467)	0.045	1.670 (1.059–2.634)	0.027
Postoperative complications (yes vs. no)	1.167 (0.766–1.778)	0.472		
Postoperative chemotherapy (no vs. yes)	0.845 (0.563–1.269)	0.417		

In univariate Cox regression, intimal invasion, age, degree of differentiation, and tumor size were significant for OS (*p* < 0.05). In the multivariate Cox regression of OS, age (HR: 2.257; 95% CI: 1.441–3.534; *p* < 0.001), tumor size (HR: 1.578; 95% CI: 1.026–2.427; *p* = 0.038), intimal invasion (HR: 2.384; 95% CI: 1.583–3.592; *p* = 0.010) were independent risk factors (Table 5).

**Table 5:** Univariable and multivariable analysis of the risk factors for OS.

Variables	Univariate analysis		Multivariate analysis	
	HR (95% CI)	*p*-value	HR (95% CI)	*p*-value
Gender (male vs. female)	1.044 (0.704–1.548)	0.832		
Age (≥60 vs. <60 years)	1.722 (1.128–2.628)	0.012	2.257 (1.441–3.534)	<0.001
History of smoking (yes vs. no)	1.183 (0.759–1.843)	0.458		
History of diabetes (yes vs. no)	0.807 (0.534–1.218)	0.307		
Albumin (<40 vs. ≥40 g/L)	0.734 (0.484–1.114)	0.146		
Total bilirubin (>26 vs. ≤26 μmol/L)	0.902 (0.606–1.343)	0.611		
CA19-9 (≥37 vs. <37 U/mL)	0.883 (0.557–1.400)	0.595		
Preoperative drainage for jaundice (yes vs. no)	1.128 (0.712–1.758)	0.608		
Blood loss (≥400 vs. <400 mL)	1.280 (0.835–1.961)	0.257		
Operation time (≥10 vs. <10 h)	0.959 (0.594–1.548)	0.864		
Degree of differentiation (poor vs. moderate-well)	1.538 (1.032–2.292)	0.035	1.253 (0.817–1.924)	0.301
Tumor size (>3 vs. ≤3 cm)	1.602 (1.071–2.397)	0.022	1.578 (1.026–2.427)	0.038
Resection margin (R1 vs. R0)	1.273 (0.658–2.463)	0.474		
Lymph node invasion (yes vs. no)	1.570 (0.985–2.502)	0.058		
Vein resection (end to end anastomosis vs. allogeneic vein)	1.014 (0.653–1.572)	0.952		
Intimal invasion (yes vs. no)	2.038 (1.373–3.023)	<0.001	2.384 (1.583–3.592)	<0.001
TNM stage (III vs. I–II)	1.341 (0.871–2.066)	0.182		
Postoperative complications (yes vs. no)	1.238 (0.828–1.853)	0.298		
Postoperative chemotherapy (no vs. yes)	0.689 (0.464–1.024)	0.066		

## Discussion

Pancreatic cancer is susceptible to early local vascular invasion and distant metastases due to its high malignancy and proximity to the portal venous system. Statistics indicate that 17 to 32% of patients demonstrate portal venous system invasion at diagnosis, whereas 50% present with distant metastasis at that time, rendering them ineligible for surgical intervention (12, 13). Siriwardana et al. examined 52 clinical studies encompassing 6,333 patients, of whom 26% exhibited venous invasion during surgery and subsequently underwent simultaneous resection and reconstruction of the involved portal vein or mesenteric vein (14). A meta-analysis of 5,242 samples revealed that 1,218 samples were pathologically proven to demonstrate tumor infiltration in the superior mesenteric vein or portal vein based on histopathological analysis following surgery, resulting in an invasion rate of 23.2% (15). For these patients, the key to surgical treatment lies in the rational and effective resection and reconstruction of the invaded veins.

Advancements in surgical procedures and perioperative management have demonstrated that pancreatic cancer resection, along with the removal and reconstruction of invaded veins, is a safe approach. In 2006, Varadhachary et al. (16) from the University of Texas MD Anderson Cancer Center first proposed the criteria for BRPC. Three years later, the American Hepato-Pancreato-Biliary Association published expert consensus guidelines on BRPC (17). Since that time, the resection of pancreatic cancer, in conjunction with vascular resection and repair, has emerged as the primary strategy for managing BRPC. A meta-analysis of 19 trials and 2,247 patients evaluated the outcomes of standard PD against PD with venous invasion resection. The findings indicated no substantial difference in perioperative complication rates or mortality rates between the two cohorts. Additionally, the overall survival rates over 5 years were comparable between the two groups of patients (OR: 0.57; 95% CI: 0.32–1.02; *p* = 0.06) (18). This further substantiates the safety of PD in conjunction with venous invasion resection and reconstruction, while also offering the potential for long-term survival for pancreatic cancer patients with portal venous system involvement. Our study additionally evaluated the long-term prognostic results of PD with concomitant venous resection in comparison to standard PD. In pancreatic cancer patients lacking pathology-confirmed venous invasion, the execution of PD with venous resection and repair did not influence their long-term survival outcomes.

From a radiological standpoint, pancreatic cancer characterized by low vascular involvement yet still suitable for surgical resection is classified as BRPC. The NCCN recommendations characterize BRPC as tumor involvement in the superior mesenteric vein-portal venous system exhibiting segmental constriction, tortuosity, or blockage (7), while yet allowing for safe reconstruction following resection. In these individuals, preoperative imaging is predominantly utilized to evaluate vascular invasion in pancreatic cancer. Previous studies suggest that the accuracy in detecting and the ability to rule out vascular invasion in PDAC using CT range from 0.58 to 0.63 and 0.92 to 0.95, respectively, whereas for EUS, the sensitivity and specificity range from 0.72 to 0.87 and 0.89 to 0.93, respectively (19–21). Despite the significantly superior sensitivity of preoperative contrast-enhanced ultrasonography (CEUS) in evaluating vascular invasion in pancreatic cancer, a notable incidence of misinterpretation persists. The proximity of inflammatory adhesions between specific arteries and tumor tissue complicates the differentiation of vascular invasion from a singular imaging viewpoint. In recent years, image-based machine learning algorithms have demonstrated significant potential in the prompt identification of pancreatic cancer (22–24), and we can similarly anticipate that machine learning will contribute to the identification of vascular invasion in pancreatic cancer.

The early clinical manifestations of pancreatic cancer lack specificity and often present as latent, nonspecific symptoms (25). These nonspecific symptoms lead to most patients being diagnosed at a borderline resectable or local advanced stage, with the tumor potentially involving major vascular structures, gradually infiltrating from the adventitia to the intima. Intimal invasion is often accompanied by the disappearance of the fat space between the pancreas and the vessel. Invasion of the muscular layer may lead to significant morphological changes in the vessels. When the tumor invades the vascular intima, it is typically associated with significant narrowing or morphological abnormalities of the portal vein or superior mesenteric vein, and may even lead to complete occlusion. Meanwhile, CD133, which is associated with cell polarity, is significantly elevated during the invasion of veins in pancreatic cancer (26–28). Patients with these characteristics generally have a poorer prognosis. In our study, we also observed that patients with pathology-confirmed venous invasion had worse overall survival and progression-free survival compared to RBC patients or BRPC with imaging-suspected venous invasion. Intimal invasion is associated with a worse prognosis compared to non-intimal invasion patients. In a retrospective study by Boggi et al. (29), involving 110 patients who underwent PD with venous resection and reconstruction for pancreatic cancer, it was observed that those with the intimal invasion of the portal venous system typically experienced a markedly poorer prognosis, with a median survival of 11 months. These findings align with the results of our research.

The findings of this study indicate that patients with confirmed pathological invasion have a somewhat unfavorable prognosis, even undergoing intensive surgical interventions that involve resection and repair of affected veins. This may arise from the significant proliferation of circulating tumor cells due to vascular invasion, complicating surgery and increasing the likelihood of postoperative recurrence and metastasis. Fundamental surgical intervention may not yield optimal therapeutic outcomes. Therefore, improving the sensitivity of preoperative imaging in assessing vascular invasion is crucial for informing the comprehensive treatment approach for these patients. The 2017 NCCN guidelines emphasize the need for neoadjuvant therapy in the treatment of non-metastatic pancreatic cancer, including borderline resectable and locally advanced cases (30). Neoadjuvant therapy provides the advantage of eliminating potential circulating tumor cells and micrometastases, hence reducing the likelihood of rapid tumor progression post-surgery and enhancing the surgical benefit. A multicenter phase III clinical trial showed that neoadjuvant sequential chemotherapy employing gemcitabine as the principal agent for resectable or borderline resectable pancreatic cancer significantly improves overall survival in patients (31). Therefore, those demonstrating confirmed vascular invasion via imaging and pathology may derive a more significant surgical benefit from neoadjuvant sequential therapy.

### Constraints of this research

The patient enrollment duration in this study was extensive, and the application of early single-slice spiral CT may have influenced the imaging assessment outcomes for certain individuals. Secondly, despite preoperative imaging evaluations of vascular invasion conducted by several imaging and pancreatic surgery specialists, a certain false positive rate persists. Third, as a single-center retrospective investigation, the sample size was comparatively limited, potentially introducing certain biases in the results. Consequently, multicenter large-sample trials are essential to further corroborate the correctness of the study findings.

## Conclusion

Pancreatic cancer patients with pathology-confirmed venous invasion have worse overall survival and disease-free survival compared to those with no invasion of the portal vein or those with imaging-suspected vascular invasion. Intimal invasion serves as an independent prognostic factor affecting postoperative survival in pancreatic cancer patients who exhibit vascular invasion.

## Data Availability

The original contributions presented in the study are included in the article/supplementary material, further inquiries can be directed to the corresponding authors.
